# Bean Leaves Ameliorate Lipotoxicity in Fatty Liver Disease

**DOI:** 10.3390/nu15132928

**Published:** 2023-06-28

**Authors:** Adriana Araceli Becerril-Campos, Minerva Ramos-Gómez, Ericka Alejandra De Los Ríos-Arellano, Perla Viridiana Ocampo-Anguiano, Adriana González-Gallardo, Yazmín Macotela, Teresa García-Gasca, Santiaga Marisela Ahumada-Solórzano

**Affiliations:** 1 Laboratory of Cellular and Molecular Biology, Faculty of Natural Sciences, Autonomous University of Queretaro, Campus Juriquilla, Av. De las Ciencias S/N, Queretaro 76230, Mexico; adi.becerril@gmail.com (A.A.B.-C.); tggasca@uaq.edu.mx (T.G.-G.); 2 Food Research and Graduate Department, School of Chemistry, Autonomous University of Queretaro, Centro Universitario, Cerro de las Campanas S/N, Queretaro 76010, Mexico; 3 Microscopy Unit, Institute of Neurobiology (INB), National Autonomous University of Mexico (UNAM), Campus UNAM-Juriquilla, Queretaro 76230, Mexico; erios.histologia.inb@gmail.com; 4 Proteogenomic Unit, Neurobiology Institute, National Autonomous University of Mexico, Campus UNAM-Juriquilla, Queretaro 76230, Mexico; gallardog@unam.mx; 5 Instituto de Neurobiología, Universidad Nacional Autónoma de México (UNAM), Campus UNAM-Juriquilla, Queretaro 76237, Mexico; macotelag@unam.mx; 6 Interdisciplinary Research in Biomedicine, Faculty of Natural Sciences, Autonomous University of Queretaro, Campus Juriquilla, Av. De las Ciencias S/N, Queretaro 76230, Mexico

**Keywords:** fatty liver, bean leaves, MAFLD, lipotoxicity, bioactive compounds, dietary fiber, polyphenols, Nrf2, PPARs, NFκB

## Abstract

Bioactive compounds in plant-based food have protective effects against metabolic alterations, including non-alcoholic fatty liver disease (NAFLD). Bean leaves are widely cultivated in the world and are a source of dietary fiber and polyphenols. High fat/high fructose diet animal models promote deleterious effects in adipose and non-adipose tissues (lipotoxicity), leading to obesity and its comorbidities. Short-term supplementation of bean leaves exhibited anti-diabetic, anti-hyperlipidemic, and anti-obesity effects in high-fat/high-fructose diet animal models. This study aimed to evaluate the effect of bean leaves supplementation in the prevention of lipotoxicity in NAFLD and contribute to elucidating the possible mechanism involved for a longer period of time. During thirteen weeks, male Wistar rats (*n* = 9/group) were fed with: (1) S: Rodent Laboratory Chow 5001^®^ (RLC); (2) SBL: 90% RLC+ 10% dry bean leaves; (3) H: high-fat/high-fructose diet; (4) HBL: H+ 10% of dry bean leaves. Overall, a HBL diet enhanced impaired glucose tolerance and ameliorated obesity, risk factors in NAFLD development. Additionally, bean leaves exerted antioxidant (↑serum GSH) and anti-inflammatory (↓mRNA TNFα in the liver) effects, prevented hepatic fat accumulation by enhanced ↑mRNA PPARα (β oxidation), and enhanced lipid peroxidation (↓liver MDA). These findings suggest that bean leaves ameliorated hepatic lipotoxicity derived from the consumption of a deleterious diet.

## 1. Introduction

Non-alcoholic fatty liver disease (NAFLD) is a metabolic hepatic disease. The disease spectrum begins in steatosis, is characterized by macrovesicular lipid accumulation (≥5% hepatocytes), and over time it progresses to steatohepatitis (NASH) with hepatocellular ballooning, oxidative stress, inflammation, and fibrosis, leading to advanced cirrhosis and hepatocarcinoma [1,2]. About 25% of the worldwide population is affected by NAFLD and its onset involves genetic factors, insulin resistance, obesity, lipotoxicity, and gut dysbiosis [2]. Lipotoxicity drives the onset and progression of NAFLD, and is the result of excessive accumulation of lipids in the liver (steatosis) and other peripheral tissues affecting their storage and oxidative capabilities. Even though steatosis is considered an early stage, it is usually accompanied by other comorbidities that can stimulate the rapid progression of the disease [3]. Steatosis is a reversible stage where the control of the comorbidities can favor its reversal and prevent the progression of the disease.

Epidemiological and experimental studies demonstrated that plant-based foods, such as common beans, (fruits, legumes, vegetables, spices, coffee, and tea) and their bioactive compounds have protective effects against steatosis, oxidative stress, inflammation, and gut dysbiosis [4]. *Phaseolus vulgaris* L. is highly worldwide consumed and cultivated because of its seeds, pods, and leaves [5]. The main nutritional compounds in bean leaves are protein (24.5–25.7%) and dietary fiber (25.9%, most of which was insoluble). Regarding polyphenols and micronutrients, bean leaves reported iron (275 mg/kg), total phenolic compounds (2.14–5.79 mg/g), and tannins (3 mg/g). Like many pulses, bean leaves had antinutritional compounds such as phytates (39.3 mg/g) and protease inhibitors (2.1 IU/mg of protein) [6,7]. Bean leaves are a low-cost alternative with nutraceutical and functional potential in human health due to their content of bioactive compounds [7].

In this regard, the effect of bean leaves supplementation in the high-fat/high-fructose feeding model was evaluated. After 8 weeks, under a 7-h daytime-restricted-feeding protocol (RFP), 10% of bean leaves supplementation improved insulin sensitivity, and diminished hepatic fat accumulation and hyperlipidemia [8]. Additionally, ad libitum bean leaves supplementation (10%) for 6 weeks without RPF prevented obesity and impairments in glucose metabolism, possibly related to an increase of 54% in SCFA (short-chain fatty acids) production [7]. However, the effect of bean leaves on oxidative stress and inflammation related to lipotoxicity in fatty liver disease has not been studied. 

Nrf2 (nuclear factor erythroid-derived 2-like 2) had aroused interest as a therapeutic target in the treatment of metabolic diseases because of its ability to regulate about 250 genes involved in the adaptive response to antioxidants and xenobiotics, under physiological and pathological conditions [9,10]. Due to Keap 1 (Kelch-like ECH-associated protein 1) sensitivity to electrophiles, mainly regulators of Nrf2, including exogenous antioxidants from plant-based food, interest has grown in understanding the role of Nrf2 as a therapeutic target for NAFLD [10]. The crosstalk between Nrf2 and PPARα (peroxisome proliferator-activated receptor alpha) promotes the oxidation of fatty acids (β oxidation) to avoid hepatic lipid accumulation [10,11]. Additionally, Nrf2 prevents the progression to steatohepatitis by modulating oxidative stress, increasing the expression of antioxidant enzymes, and by delaying the inflammatory response mediated by NFκB (nuclear factor kappa B) [10,12].

Based on the reported beneficial effects of bioactive compounds and the interest in improving people’s health, through the revaluing and encouraging the inclusion of vegetables in the diet such as highly cultivated bean leaves, this study aims to evaluate the effect of 10% bean leaves dietary supplementation to prevent lipotoxicity in fatty liver disease and related comorbidities as obesity, insulin resistance, and hyperlipemia.

## 2. Materials and Methods

### 2.1. Diet Design

Bean leaves (*Phaseolus vulgaris* L.) from the Flor Mayo Eugenia variety were cropped after 60–70 cultivating days at the experimental campus of the Autonomous University of Queretaro, Amazcala, Mexico. Bean leaves were dried at 40 °C (to constant weight), ground, and stored in darkness at room temperature (RT), to further analyze their chemical composition. Based on the proximal analysis outcome, four diets were designed (Table 1):

(1) S: Rodent Laboratory Chow 5001^®^ (RLC), 3.4 kcal/g;
(2) SBL: mixture of 90% RLC+ 10% dry bean leaves, 3.6 kcal/g;
(3) H: high fat (lard) and high fructose diet, 4.4 kcal/g;
(4) HBL: H was supplemented with 10% of dry bean leaves, 4.6 kcal/g [7].

### 2.2. Experimental Design

The experimental design and procedures were previously approved by the Ethics Committee of the Faculty of Natural Sciences, Autonomous University of Queretaro (77FCN2019); and the number of rats was minimized following the 3Rs principles [13]. 

Thirty-six male Wistar rats (198.4 ± 1.6 g) were aleatory randomized after one week of acclimatization in four experimental groups: (1) S, (2) SBL, (3) H, and (4) HBL. The rats were housed in individual plastic cages for thirteen weeks, kept under fully controlled conditions (temperature and moisture), 12/12 h dark/light cycle, with water and food ad libitum. After 13 weeks, blood and liver samples were collected, processed, and maintained at −80 °C until their analysis.

### 2.3. Body Measurements and Body Composition

Body weight, food intake, and water intake were registered weekly. At the thirteenth week, body length, abdominal (AC) and thoracic circumferences (TC), AC/TC ratio, and body mass index (BMI) [14] were measured in awake, unanesthetized rats. Total body weight gain was calculated as the difference between the final and the initial body weight. The energy and food intake per day are the averages of the week’s registration, and the total energy and total food intake are cumulative sums of the whole period.

In the last experimental week, five animals from each group were selected to perform magnetic resonance imaging (MRI). The rats were anesthetized with 1.5–2% isoflurane in combination with medical air. MRI analyses were performed using Teslas Bruker Pharmascan 70/16US MR scanner (MA, USA) in the National Laboratory for magnetic resonance imaging (LANIREM-INB-UNAM). The image was acquired by water-suppressed Turbo Rapid Imaging with Refocused Echos in two dimensions (RARE, Rapid Imaging with Refocused Echoe factor = 8). The scanning time was 1:40 min with motion compensation by respiratory-triggered acquisition and considering the following parameters: matrix size 209 × 191, 54 slices with 3 mm thickness and slice gap 2 mm, repetition time 4359 ms, echo time 25 ms, and 5 averages. The field of view was 65 × 47 mm, with a resolution of 311 × 247 μm and a flip angle of 90°. Coronal and transversal images were used for fat quantification [3]. Fat quantification was performed by segmentation using the 3D Slicer^®^ program [15,16].

### 2.4. Analysis of Biochemical Parameters

Postprandial glucose tolerance was evaluated during the thirteenth week by an intraperitoneal glucose tolerance test (IpGTT) on 4–6 h fasting rats. Baseline glycemia (0 min) was measured in the caudal vein with the Accu-Check Active^®^ glucometer (Chennai, India). Intraperitoneal injection of 20% glucose solution (2 g/kg) was administrated and glycemia was measured at 30, 60, 90, and 120 min [17]. 

Enzymatic-colorimetric assays were used to determine circulating glucose, total cholesterol, triglycerides, high-density lipoprotein cholesterol (HDL-c), and low-density lipoprotein cholesterol (LDL-c) parameters. In order to evaluate liver function, the levels of aspartate aminotransferase (AST), alanine aminotransferase (ALT), albumin (A), and globulin (G) were determined. All the analyses were carried out in serum samples by using Spinreact^®^ reagents in the clinical analyzer Spin 120^®^ [7]. 

Insulin (ELISA Rat insulin kit Alpco^®^ 2820242), oxidized LDL (Rat Ox-LDL ELISA kit MYBioSurce MBS2501477), and C-reactive protein (Rat CRP SimpleStep ELISA Kit Abcam, ab256398) were analyzed by immunoassay on a microplate reader (Molecular Devices Spectramax 250). AST/ALT ratio, A/G ratio (albumin/globulin), very-low-density lipoprotein (VLDL-c), and the triglycerides/ HDL-c ratio were calculated. Homeostasis model assessment of insulin resistance (HOMA-IR) and homeostasis model assessment of pancreatic β cell function (HOMA-β) were estimated considering fasting levels, with the following equations [18,19]:

HOMA-IR = [insulin (μIU/mL) × glucose (mmol/L)]/22.5

HOMA-β = [20 × insulin (μIU/mL)/glucose (mmol/L) − 3.5]

### 2.5. Macroscopic and Microscopic Liver Examination

After the animals were euthanized, each liver was removed, weighed, and examined. Left lateral lobe samples were taken for further analysis. For histological analysis samples were fixed in phosphate-buffered 10% formalin. Paraffin-embedded sections were sliced (5 µm) and stained with hematoxylin and eosin (H&E). Histological evaluation was performed following Brunt’s scoring system, under 400× magnification at Velab (VE-BC3PLUS) microscope [20,21].

### 2.6. Liver Triglyceride and Antioxidant Enzyme Determination

Liver samples (0.3 g) were pulverized using liquid nitrogen. For liver triglyceride extraction Folch reagent (2 chloroform: 1 methanol) was added to the pulverized tissue (20:1 *v*/*w*). Samples were vortexed for 3 min at RT and sonicated for 20 min at 4 °C. To induce phases separation, NaCl 0.9% (1:5 *v*/*v*) was added to each sample and samples were centrifuged (1000× *g* × 10 min at 4 °C). The remaining chloroform/methanol/water phase was evaporated from the lower phase to get a lipid pellet. Once the pellet was reconstituted (NaCl 0.9%), triglycerides were measured by enzymatic-colorimetric assay (Spinreact^®^ reagent, Catalonia, Spain) [7,22,23].

For lipid peroxidation, 25 mg of liver were homogenized with 250 µL of RIPA buffer and centrifuged 1600× *g* × 10 min at 4 °C. Malondialdehyde (MDA 36357, Merck, Darmstadt, Germany) was used as standard (0, 0.625, 1.25, 5, 25, 75 µM). Thiobarbituric acid (TBA 10%), sodium hydroxide (3.5 M), and trichloroacetic acid (10%) were added to the samples and standards. After boiling (90–100 °C) for 1 h, MDA-TBA adducts were formed and measured at 540 nm on a microplate reader (Molecular Devices Spectramax 250, Marshall Scientific, NH, USA) [24].

For glutathione (GSH), 500 mg were homogenized with 3 mL of Tris-sucrose buffer (pH 6.5) and centrifuged (8000× *g* × 20 min at 4 °C). For the preparation of the cytosolic supernatant serum and liver samples were ultracentrifuged (100,000× *g* × 1 h at 4 °C) and precipitated with TCA (20%) to get concentrated cytosols. GSH determination was performed by Ellman’s method and GSH reagent (Sigma Aldrich PHR1359, Darmstadt, Germany) was used as standard. After 5 min of incubation at RT, the 96-well plate was read on a Varioskan LUX Multimode Microplate Reader (Thermo Fisher Scientific, MA, USA) at λ 412 nm [25].

Liver samples (500 mg) were homogenized with PBS (50 mM, pH 7) and centrifuged (8000× *g* × 20 min at 4 °C) to obtain the cytosolic supernatants for antioxidant enzymes measurements. Catalase (CAT) activity was determined by Aebi´s method, using 30 mM of H_2_O_2_. Optical density absorbance measurements were recorded for 30 s (6 × 5 s) at 240 nm [26]. Glutathione peroxidase (GPx) activity was analyzed with Glutathione Peroxidase Assay Kit (Merck 353919, Darmstadt, Germany). The decreasing rate in the absorbance (340 nm) is directly proportional to the oxidation of NADPH to NADP+ [27]. Protein concentrations in liver homogenates were quantified by the bicinchoninic acid (BCA) assay (Thermo Fisher Scientific 23227, MA, USA), using BSA (bovine serum album) as a standard.

### 2.7. Expression Analysis

Total RNA from liver samples was isolated employing the TRIzol reagent (Invitrogen 15596026) [28]. RNA integrity was evaluated by electrophoresis and its concentration was determined by spectrophotometric analysis (NanoDrop 1000, Wilmington, DE, USA). To be able to amplify and quantify the RNA expression, cDNA was synthesized by reverse transcription reaction. To unwind RNA, 1 µg of total RNA was heated at 70 °C for 5 min. First, 1 µL of each of the following reagents were mixed and preincubated for 2 min at 42 °C, heat-denatured RNA, antisense oligonucleotides (15 bases synthesized by IDT), dNTP (Thermo Fisher Scientific R0181, MA, USA R0181), random primers (Promega C1181). Then, reverse transcriptase (Promega M1701) was added and the mixture was heated at 70 °C for 15 min. cDNA was kept at −20 °C and used to quantify gene expression of *Tnfa*, *Nfe2l2*, *Ppara,* and *Hmox1* (Table 2) by quantitative PCR (qPCR) analyses in the LightCycler^®^ 2.0 instrument with the LightCycler FastStart DNA Master Sybr Green I kit (Roche Applied Science, Mannheim, Germany). *Sod2* and *Ywhaz* were used as housekeeping genes after their validation by NormFinder [29]. Gene expressions were analyzed by the 2^−ΔΔCT^ method [30]. Amplicon identity was corroborated by sequences and BLAST (NIH) analysis. 

Liver sections, previously paraffin-embedded and sliced (5 µm), were dewaxed and rehydrated. After antigen retrial with HCl 2 M for 30 min and permeabilization, the samples were incubated for 2 h with normal goat serum (1:20) for blocking [31]. Slices were incubated for 24 h with primary Nrf2-antibody (Abcam 89443, Cambridge, UK) and PPARα (Abcam215270, Cambridge, UK). To avoid non-specific fluorescence, a 15 min incubation with Sudan Black B (0.1%) was performed [32]. After 12 h of secondary antibody incubation (Abcam150113 and 150077, Cambridge, UK), slices were stained with DAPI (Sigma-AldrichSLCB0123, Darmstadt, Germany). Nine images per slice were captured on an Apotome Zeiss microscope. Pearson’s correlation coefficient (PCC) and Mander’s correlation coefficient (MCC) were performed to evaluate colocalization [33] with the image analyzer Fiji [34].

### 2.8. Statistical Analysis

The data are presented as mean± standard error of the mean (SEM). The Shapiro–Wilk test was executed in the continuous variables to assess their normal distribution. In order to know the similarity of SBL and HBL to the S group and describe the NAFLD model, statistical analysis was performed by one-way ANOVA, and the differences against the control group (S) were analyzed by Dunnet’s post hoc test, * *p* ≤ 0.05. Further, to determine the preventive effect of bean leaves supplementation in a high-fat and high-fructose model, the statistical differences between H and HBL groups were analyzed by Student’s *t*-test, # *p* ≤ 0.05. Graphics were carried out with GraphPad Prism 8 (Dotmatics, San Diego, CA, USA), and SPSS Statistics v.25 (IBM, NY, USA) was used for the statistical analysis.

## 3. Results

### 3.1. Effect of Bean Leaves on Body Fat Accumulation

The rats were fed with a standard diet (S) and simultaneously supplemented with 10% of bean leaves (SBL). Meanwhile, experimental rats were fed with a high-fat/high-fructose diet (H) to induce obesity and hepatic lipotoxicity; and in order to prevent these metabolic alterations another group was supplemented with 10% of bean leaves (HBL). 

**Figure 1:**
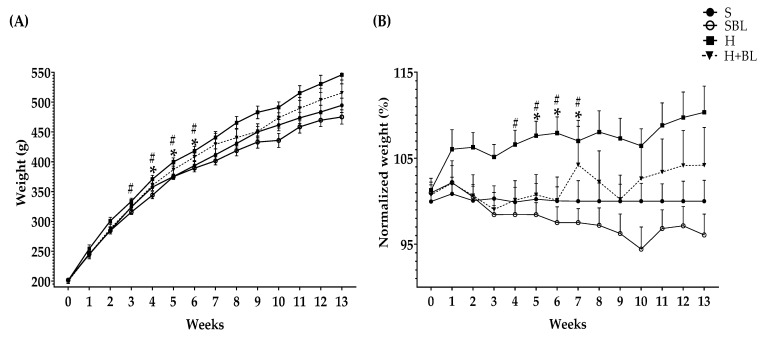
Effect of common bean leaves in (**A**) weight per week and (**B**) normalized weight % against the correspondent weight of standard diet group or S-diet group. Values represent the mean ± SEM (*n* = 9). ANOVA post hoc Dunnet’s test was performed to compare groups versus S * *p* ≤ 0.05. Student’s *t*-test was performed to compare H versus HBL # *p* ≤ 0.05. S = standard diet, SBL = S + 10% bean leaves, H = high-fat/high-fructose diet, HBL = H + 10% bean leaves.

**Figure 2:**
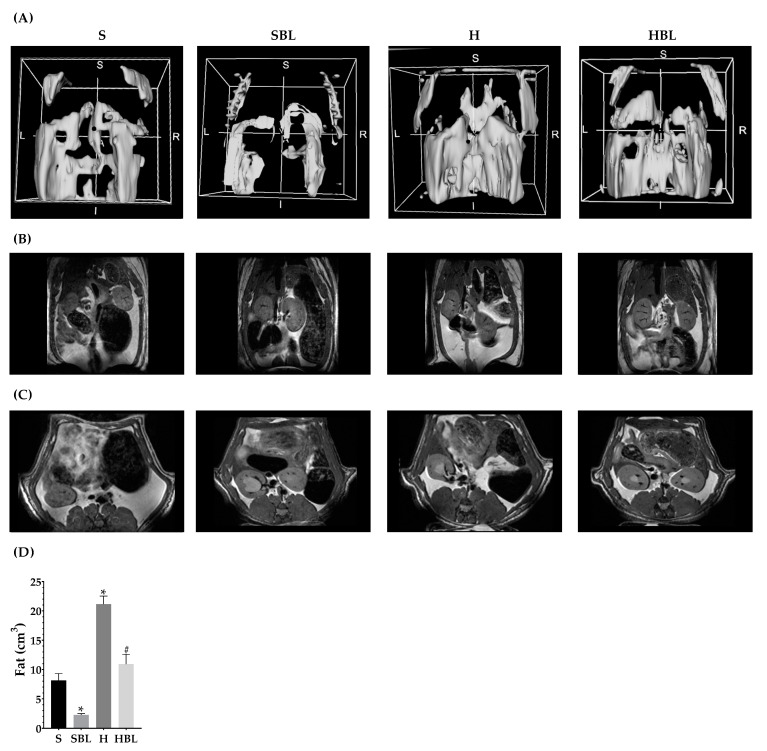
Effect of common bean leaves in body fat accumulation. Body fat accumulation was evaluated by magnetic resonance (**B**) in coronal and (**C**) in transversal plane. Analysis was performed on 3D Slicer^®^, (**A**) shows a 3D reconstruction, and (**D**) fat quantification. Values represent the mean ± SEM (*n* = 5). ANOVA post hoc Dunnet’s test was performed to compare groups versus S * *p* ≤ 0.05. Student’s *t*-test was performed to compare H versus HBL # *p* ≤ 0.05. S = standard diet, SBL = S + 10% bean leaves, H = high-fat/high-fructose diet, HBL = H + 10% bean leaves.

Body weight was measured weekly (Figure 1). The main statistical differences in weekly body weight were between weeks 4 to 6 (Figure 1), H gained higher weight (↑4–6%), compared to that of the S group. After 13 weeks, total body weight ↑8% (Table 3) and body fat accumulation by MRI ↑160% (Figure 2) in the H group were higher than those of the S group; due to ↑30% total energy intake and 32% in daily energy intake (Table 4). Water intake total food intake (Table 4) and body measurements as length, AC, TC, AC/TC ratio, or BMI (Table 3) in the H group do not have differences compared to the S group. H rats developed obesity, by the increase in energy intake, body weight gain, and body fat accumulation.

Interestingly, the supplementation with 10% of bean leaves (HBL) lead to ↓2–4% less body weight between weeks 3 and 7 and ↓48% decreased body fat accumulation by MRI (Figure 2) at the end of the 13 weeks, ↓7% reduced thoracic circumference and ↓5% shorter length (Table 3), compared to H. Food, water, or energy intake, and AC, AC/TC ratio, or BMI (Table 4) in HBL rats did not show differences to those in H group. Even though the HBL group showed less water intake (total and daily) compared to the S group, the water intake of all groups was in the recommended range (8–12 mL of water/100 g body weight) [35,36].

Additionally, HBL showed a protective role against obesity development (↓body fat accumulation by MRI) in spite of total energy intake being similar to H and ↑38% more than S (*p* ≤ 0.05). Additionally, the supplementation with 10% of bean leaves in the standard diet (SBL) reduced ↓72% of body fat accumulation by MRI (Figure 2), without evidence of a possible negative effect of bean leaves intake for 13 weeks.

### 3.2. Effect of Bean Leaves on Insulin Resistance, Impaired Glucose Tolerance, and Dyslipidemia

Alteration in the oxidative and storage capacity of lipids in peripheral tissues (lipotoxicity) due to excessive fat accumulation [3] has an important role in insulin resistance and an increase in circulating lipids [37]. Therefore, we analyzed fasting levels of lipids, glucose, and glucose tolerance in the rats after thirteen weeks. Fasted glucose levels (Table 5) showed by all groups were normal [38]. Moreover, fasted glucose level (Table 5) in the H group was similar to that in the S group, but fasted insulin was higher (↑142%) in H rats. These results suggested compensatory hyperinsulinemia in H animals, related to the increase in the pancreatic β-cell function (HOMA-β ↑259%) due to a diminished capacity of the tissues to utilize insulin (HOMA-IR ↑116%) compared to S.

**Figure 3:**
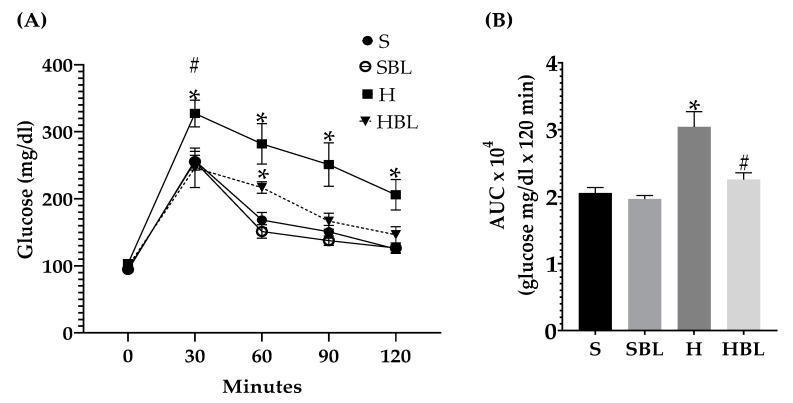
Effect of common bean leaves in (**A**) postprandial glycemic response and (**B**) area under the curve in intraperitoneal glycemic tolerance test. Values represent the mean ± SEM (*n* = 9). ANOVA post hoc Dunnet’s test was performed to compare groups versus S * *p* ≤ 0.05. Student’s *t*-test was performed to compare H versus HBL # *p* ≤ 0.05. S = standard diet, SBL = S + 10% bean leaves, H = high-fat/high-fructose diet, HBL = H + 10% bean leaves, AUC = area under the curve.

Besides during IpGTT, postprandial glycemia (Figure 3A) in H rats rose ↑27% at 30 min and ↑65% at 60, 90, and 120 min, and glycemic global response (AUC, Figure 3B) increased ↑48%, compared to those of S rats. This impaired glucose tolerance and compensatory hyperinsulinemia in the H animals was accompanied by hyperlipidemia (Table 6). Circulating lipids increased in the H group, ↑26% in total cholesterol, ↑58% in triglycerides, ↑58% in VLDL-c, and ↑17% HDL-c, compared to those of the S group.

Fasted glucose of HBL rats was similar to that of the H group, but insulin was lower ↓22% (without statistical differences). However fasted insulin of HBL increased ↑88%, but glucose, HOMA-IR, and HOMA-β (Table 5) did not show statistical differences against those of the S group, leaving HBL in a middle point between H and S, without effects on fasting glucose.

Meanwhile, in postprandial glucose metabolism (Figure 3), during IpGTT, glucose tolerance improved (↓26%AUC) in HBL rats compared to H rats. Moreover, the similarity of the shape of the curve, resulting from IpGTT, between the HBL curve to S and SBL curves could suggest an improved glucose metabolism [39].

Regarding circulating lipids, total cholesterol (↓11%) was lower in the HBL group compared to the H group (Table 6), these could be associated with less accumulation of body fat. Bean leaves supplementation (HBL group) did not show an effect on hypertriglyceridemia (↑41%) and increased VLDL-c (↑41%) compared to S levels. The data suggested that bean leaves supplementation in a high-fat/high-fructose diet (HBL) improved insulin resistance without increasing β pancreatic cells function, and enhanced impaired glucose tolerance.

### 3.3. Effect of Bean Leaves on the Silent Stage of NAFLD, Steatosis

**Figure 4:**
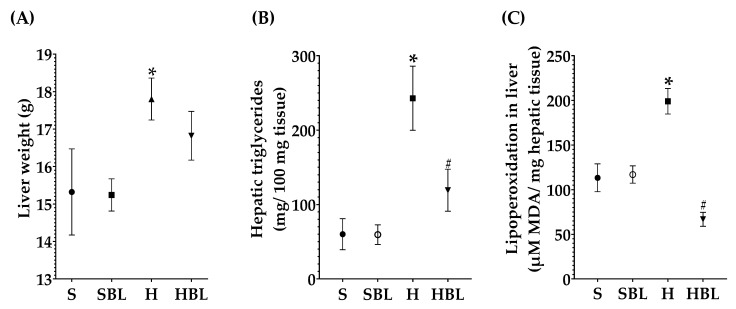
Effect of common bean leaves in (**A**) hepatic weight, (**B**) triglycerides quantification, and (**C**) lipid peroxidation. Values are mean ± SEM (*n* = 9), comparison against S was analyzed by ANOVA post hoc Dunnet * *p* ≤ 0.05, comparison between H and. HBL was analyzed with Student’s *t*-test # *p* ≤ 0.05. S = standard diet, SBL = S + 10% bean leaves, H = high-fat/high-fructose diet, HBL = H + 10% bean leaves, MDA = malondialdehyde.

**Figure 5:**
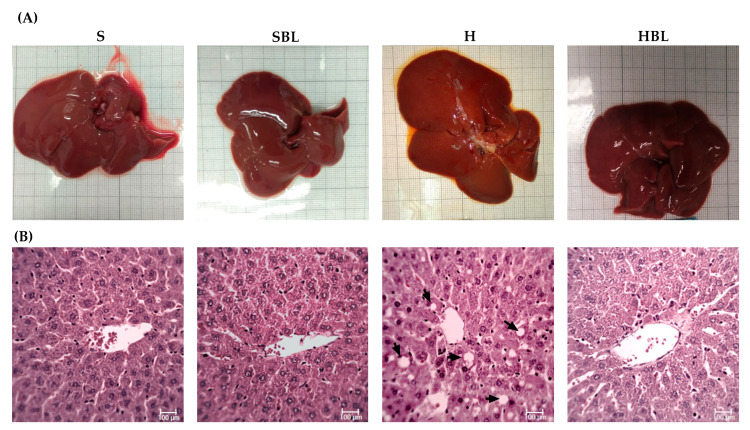
Effect of common bean leaves in (**A**) macroscopic appearance of liver and (**B**) histoarchitecture of hepatic tissue by H&E 400×. The arrows indicate lipid droplets inside the hepatocytes. S = standard diet, SBL = S + 10% bean leaves, H = high-fat/high-fructose diet, HBL = H + 10% bean leaves, H&E = hematoxylin and eosin.

Besides the effect of bean leaves preventing obesity and insulin resistance, metabolic alterations that play a key role in NAFLD development, we further explore the effect of bean leaves in steatosis, the earliest stage of NAFLD. As expected, the H diet promoted fatty liver accumulation and lipoperoxidation, as evidenced by the increase in hepatic triglycerides accumulation ↑303%, liver weight ↑16%, and MDA ↑75% (Figure 4), compared to those of the S diet. The increase in hepatic triglycerides matches with the macroscopic appearance of the liver, pale red color with yellowish spots (Figure 5), and the histopathological analysis, macrovesicular steatosis (<33%) in the centrilobular zone (Table 7), without loss of hepatic zonation (Figure 5). H rats developed steatosis grade I (Table 7, Figure 5) without changes in liver function serum parameters and neither in protein C reactive (Table 8).

**Figure 6:**
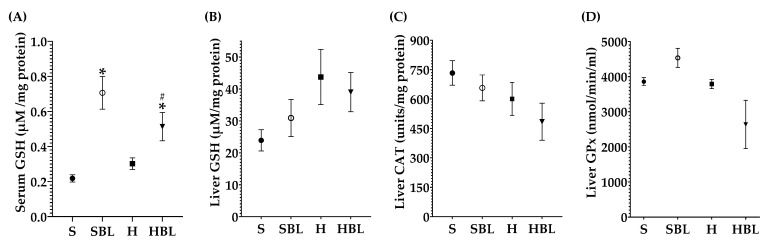
Effect of common bean leaves in antioxidant enzymes: (**A**) reduced glutathione (GSH) in serum, (**B**) GSH in hepatic tissue, (**C**) catalase (CAT) quantification in liver, and (**D**) glutathione peroxidase (GPx) in liver. Values are mean ± SEM (*n* = 9), comparison against S was analyzed by ANOVA post hoc Dunnet * *p* ≤ 0.05, comparison between H and. HBL was analyzed with Student’s *t*-test # *p* ≤ 0.05. S = standard diet, SBL = S + 10% bean leaves, H = high-fat/high-fructose diet, HBL = H + 10% bean leaves.

**Figure 7:**
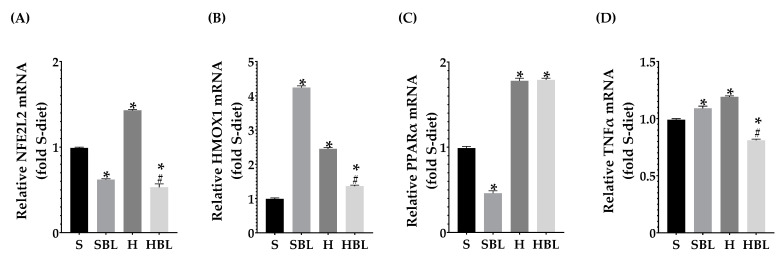
Effect of common bean leaves in relative mRNA expression of (**A**) *Nfe2l2*, (**B**) *Hmox1*, (**C**) *Ppara,* and (**D**) *Tnfa*. RT-qPCR analyzed by Livak’s method; *Sod2* and *Ywhaz* were used for normalization as housekeeping genes. Values are mean ± SEM (*n* = 9), comparison against S was analyzed by ANOVA post hoc Dunnet * *p* ≤ 0.001, comparison between H and. HBL was analyzed with Student’s *t*-test # *p* ≤ 0.0001. S = standard diet, SBL = S + 10% bean leaves, H = high-fat/high-fructose diet, HBL = H + 10% bean leaves, *Sod2* = Superoxide dismutase 2, *Ywhaz =* Tyrosine 3-monooxygenase/tryptophan 5-monooxygenase activation protein zeta, *Tnfa* = Tumor necrosis factor-alpha, *Nfe2l2 =* Nuclear factor erythroid 2-related factor 2, *Ppara* = Peroxisome proliferator-activated receptor alpha, *Hmox1* = Heme oxygenase 1.

Even though there were no statistical differences in liver antioxidant enzymes activity between the H and S rats (Figure 6), there are some changes that could have biological importance; as a defense mechanism in the H group GSH increased ↑83% in the liver and ↑38% in serum (Figure 6A,B) compared to those of the S group, trying to keep the redox homeostasis. Meanwhile, *Hmox1* mRNA expression increased ↑147% (Figure 7B) and *Tnfa* mRNA ↑20% (Figure 7D) in the H rats, compared to those of the S rats. The findings suggested that the livers of our high-fat/high-fructose animals (H group) had lipid peroxidation and inflammation.

**Figure 8:**
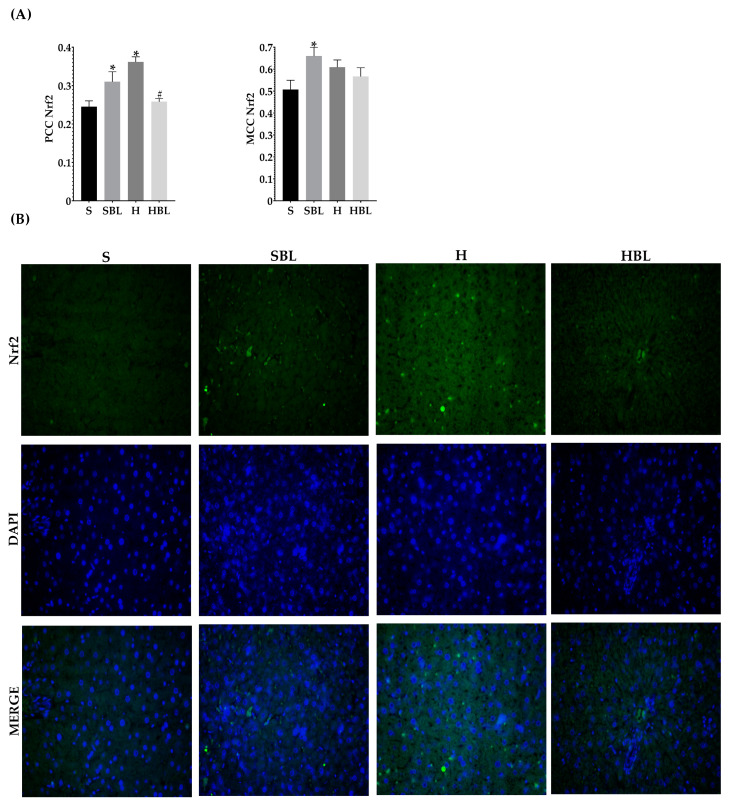
Effect of common bean leaves in Nrf2 nuclear translocation. (**A**) Graphs shows colocalization coefficients for Nrf2 in the nucleus: Pearson’s correlation coefficient (PCC), Mander’s correlation coefficient (MCC). (**B**) Immunofluorescence staining for Nrf2 (green Alexa488) and nucleus (blue, DAPI). Values are mean ± SEM (*n* = 5, 9 photos per slice), comparation against S was analyzed by ANOVA post hoc Dunnet * *p* ≤ 0.001, comparison between H and. HBL was analyzed with Student’s *t*-test # *p* ≤ 0.0001. S = standard diet, SBL = S + 10% bean leaves, H = high-fat/high fructose diet, HBL = H + 10% bean leaves, Nrf2 = nuclear factor erythroid 2-related factor 2.

**Figure 9:**
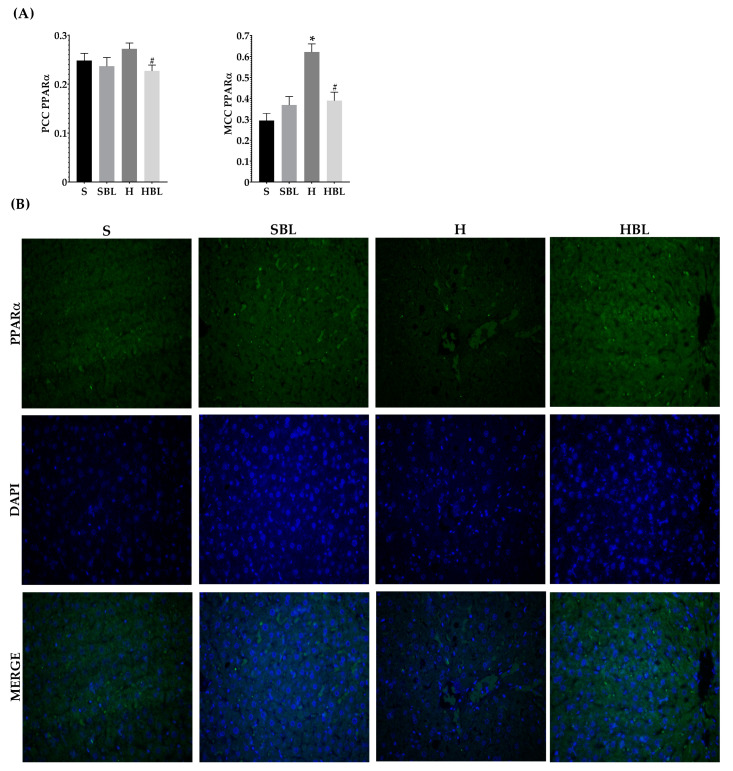
Effect of common bean leaves in PPARα nuclear translocation. (**A**) Graphs show colocalization coefficients for PPARα in the nucleus: Pearson’s correlation coefficient (PCC), Mander’s correlation coefficient (MCC) (**B**) Immunofluorescence staining for PPARα 2 (green Alexa488) and nucleus (blue, DAPI). Values are mean ± SEM (*n* = 5, 9 photos per slice), comparison against S was analyzed by ANOVA post hoc Dunnet * *p* ≤ 0.001, comparison between H and. HBLwas analyzed with Student’s *t*-test # *p* ≤ 0.0001. S = standard diet, SBL = S + 10% bean leaves, H = high-fat/high-fructose diet, HBL = H + 10% bean leaves, PPARα = Peroxisome proliferator-activated receptor alpha.

Moreover, the H diet increased *Nfe2l2* mRNA expression ↑44% (Figure 7A) and increased the signal intensity of Nrf2 nuclear localization ↑48% (Figure 8), these could be due to the development of steatosis grade I, insulin resistance, and obesity in H group.Moreover, *Ppara* mRNA expression increased ↑80% (Figure 7C) and PPARα nuclear translocation ↑112% (Figure 9) were higher in H rats compared to those of the S group, as expected in murine steatosis models, where *Ppara* overexpression is a defense mechanism of the liver to delay NAFLD progression to NASH [40]. 

Therefore, the data showed that our high fat/ high fructose animals (H group) had obesity, insulin resistance, impaired glucose tolerance, and hyperlipidemia, and as a consequence of those, the livers of H animals developed steatosis I, lipid peroxidation, and inflammation. Because steatosis is an early stage of NAFLD, the livers of H rats kept defense mechanisms against fat accumulation, inflammation, and oxidative stress.

On the other hand, bean leaves supplementation (HBL) in a high-fat/high-fructose diet has shown a protective effect against impaired glucose tolerance, insulin resistance, and hypercholesterolemia. After 13 weeks, hepatic triglycerides were ↓51% lower in HBL rats than that of the H group (Figure 4), interestingly without any statistical difference against the S group. Hepatic triglycerides levels in HBL rats match not only with the histopathological analysis, macroscopically the liver had an intense red color without yellowish pots (Figure 5), and microscopically the liver had centrilobular microvesicular fat accumulation < 5%, without steatosis (Table 7), but also with the absence of liver function alterations (Table 8). Moreover, SBL animals did not exhibit any hepatic damage after thirteen weeks of supplementation with 10% of bean leaves.

Moreover, MDA hepatic levels in the HBL group decreased ↓66%, showing a protective effect against lipid peroxidation that was present in the H group. Even though liver CAT and GPx activity had not shown any statistical differences between groups (Figure 6), neither did hepatic GSH levels; physiologically, they had decreased. CAT activity (↓24%), GPx activity (↓66%), and hepatic GSH (↓11%) were lower in HBL compared to H (Figure 6), probably because of less generation of hydroperoxides (↓MDA levels).

Interestingly, serum GSH levels in HBL animals increased ↑70% compared to that of the H animals and ↑136% compared to that of the S rats. In addition, serum GSH levels in SBL rats incremented ↑224% (Figure 6), compared to that of the S rats. Moreover, *Hmox1* mRNA expression drop ↓45% in HBL rats compared to H, possibly due to a lower fat accumulation and lipid peroxidation. However, *Hmox1* mRNA expression rose ↑37% in the HBL group and ↑328% times in the SBL group (Figure 7B), compared to that of the S group. These findings suggested that bean leaf supplementation enhanced antioxidant capabilities in the liver.

After 13 weeks (Figure 7A), possibly owing to the rise of GSH levels in serum, fat accumulation, and lower lipid peroxidation in the liver, HBL rats exhibited reduced expression of *Nfe2l2* mRNA ↓63% and Nrf2 nuclear intensity decreased ↓29% (PCC), compared to those of H rats. Meanwhile, *Nfe2l2* mRNA expression in HBL decreased ↓46% compared to S; but in the SBL group the intensity and translocation to the nucleus rose ↑27% for PCC and ↑27% for MCC (Figure 8), even though when the expression was ↓37% lower.

Regarding PPARα which is a main regulator of lipid metabolism, livers from HBL animals showed increased expression of Ppara mRNA ↑80% (Figure 7C) compared to that of S animals. The overexpression of PPARα could protect the liver against fat accumulation and lipid peroxidation [41]. However, the intensity and nuclear translocation in the HBL group diminished (Figure 9), ↓16% (PCC) and ↓37% (MCC) respectively, compared to the S group. Moreover, SBL rats showed reduced expression of Ppara mRNA ↓53% and ↓81% (MCC) lower translocation to the nucleus, compared to those of S rats. These lower expressions of PPARα in SBL could be related to a lower content of fatty acids in the liver. Appealingly, HBL rats exhibited reduced expression of Tnfa mRNA ↓ compared to H, and ↓ compared to S (Figure 7D).

Therefore, bean leaves showed ameliorated hepatic lipotoxicity derived from the consumption of a deleterious diet by a protective effect against inflammatory alterations and an antioxidant protector. Along with enhanced insulin resistance without increasing β pancreatic cells function, they enhanced impaired glucose tolerance.

## 4. Discussion

Bean leaves are a source of bioactive compounds like iron, protein, insoluble fiber, and polyphenols. Bioactive compounds of plant-based foods have been shown to play a role in the prevention of metabolic disorders related to high-fat/high-fructose diets [7,8]. The 10% of bean leaves supplementation that has shown a protective effect against lipotoxicity in fatty liver disease (12.6 g/kg body weight), extrapolated to humans the dose should be 2 g/kg of body weight [42]. This dose represents 30% of the daily recommended intake of fruits and vegetables (400 g per day) to prevent chronic diseases [43].

A positive energy balance coming from a high energy intake promotes excessive accumulation of visceral fat and obesity-related comorbidities [44,45]. As expected, the H group showed obesity (↑body weight gain and ↑body fat accumulation by MRI), as reported in high-fat/high-fructose diet models [45]. Meanwhile, HBL prevented obesity (↓body fat accumulation by MRI without changes in body weight gain). Rats had shown compensatory mechanisms in weight change, probably due to this HBL didn’t show differences in weight gain against either S or H groups [46,47], further analysis is needed to evaluate the effect of bean leaves in the hypothalamic control center.

Previous data suggested that the decrease in body fat accumulation and weight gain after the supplementation with 10% of bean leaves in a high-fat/high-fructose diet, may not be due to alterations in fat absorption or fat fecal excretion [7]. Further studies are required to evaluate the role of bean leaves supplementation for longer time periods and to understand if bean leaves have a role in decreased fat absorption or increased thermogenesis, based on both high-fat/high-fructose groups (H and HBL) had similar energy intake but HBL showed less body fat accumulation.

High-fat/high-fructose diet intake drives insulin resistance adipocytes, leading to an increase in free fatty acids (FFAs) flux duet to increased lipolysis in adipose tissue [48,49]. Therefore, FFAS flux promotes deleterious effects in non-adipose tissue [3]. In the liver, FFAs raise the synthesis of triglyceride-enriched VLDL-c, which also generates LDL-c and HDL-c triglyceride-enriched. These HDL-c are easily cleared by the kidney, resulting in few HDL-c being able to accept cholesterol from the vasculature [49]. Additionally, patients with NAFLD had shown increased persistent VLDL secretion due to an overexpression of MTP (microsomal triglyceride transfer protein) [50]. Hence, the H group developed obesity and dyslipidemia by increased total cholesterol, triglycerides, VLDL-c, and HDL-c, in addition to impaired glucose tolerance, compensatory hyperinsulinemia, and insulin resistance. These common metabolic alterations induced by high-fat/high-fructose models are strongly related to nonalcoholic liver disease development [45].

Meanwhile, 10% bean leaves supplementation (HBL) prevented impaired glucose tolerance, hypercholesterolemia, and improved insulin resistance due to less body fat accumulation. HBL-diet had similar fasting glucose, HOMA-IR, and HOMA-β levels to the S-diet, but without differences with the H-diet; meanwhile, the postprandial glucose level in the HBL group was lower than that in the H group. Fasted glucose levels could be increased by the high-fructose diet [51]; this could be related to no differences between the H and the HBL groups. Additionally, the liver is mainly responsible for fasting glucose levels and the pancreas is responsible for postprandial glucose levels; therefore, the effect of bean leaves in β cell pancreatic function needs further investigation [51].

In a previous study, where bean leaves supplementation with 7-h daytime RFP was evaluated, fasting glucose levels and insulin resistance (HOMA-IR) were lower after 8 weeks of treatment [8]. Dietary fiber and phenolic compounds present in bean leaves had shown an increase in SCFA production on cecal content [7]. SCFA are synthesized by microbiota bacteria such as *Bifidobacterium* and *Lactobacillus*. Binding of SCFAs to their free fatty acid receptors (FFAR2/FFAR3) on enteroendocrine cells results in stimulated secretion of glucagon-like peptide 1 (GLP-1) that promotes insulin secretion and peptide YY (PYY) which reduces food intake. Meanwhile, in pancreatic β-cells, the interaction between SCFA and FFAR2/FFAR3 promotes insulin secretion [52]. Likewise, butyrate increases the expression of phosphoenolpyruvate carboxykinase-1 and glucose-6 phosphatase, key enzymes in intestinal gluconeogenesis sensed by the portal vein, improving insulin sensitivity [53]. This could be a possible mechanism of action of bean leaves on glucose metabolism that should be further explored.

Even when previous studies with bean leaves supplementation in high-fat/high-fructose diets had not shown decreases in circulating lipid levels [7,8,54], bean leaves supplementation with RFP downregulated *Scd1* (stearyl-coenzyme A desaturase1) expression in liver [8], leading to triglycerides exportation, keeping high levels of VLDL-c and triglycerides in circulation [54]. As with other plant-based diet interventions, bean leaf intake should be evaluated together with physical activity and changes in lifestyle [55].

Insulin resistance increases hepatic gluconeogenesis; likewise, compensatory hyperinsulinemia raises de novo lipogenesis [56,57]. The H animals not only showed compensatory hyperinsulinemia and insulin resistance, keeping similar glucose lower to the S group with higher levels of insulin, but also an increased β pancreatic function. Additionally, the increased FFAs flux downregulates β oxidation by the PPARα pathway (proliferator-activated receptor alpha) [58]. This disbalance between the synthesis of triglycerides and the capacity to utilize and export them (VLDL-c synthesis), saturates the liver capacity, leading to hepatic lipotoxicity [48,59]. Hepatic lipotoxicity has a key role in the progression of NAFLD, and it is present since its first stage (steatosis) [59]. As part of this process, H rats developed steatosis grade I, liver of H rats preserve protective mechanisms against fat accumulation, such as hepatic exportation of triglycerides (↑VLDL) and fatty acid oxidation facility (↑Ppara).

Steatosis is characterized by accumulating fat in up to 5% of the hepatocytes, and lipid peroxidation, without fibrosis, ballooning, inflammation, or changes in serum liver function parameters [59,60]. High fat and fructose models induce alterations in glucose and lipid metabolism, such as insulin resistance and an increase in the circulating lipids, strongly associated with oxidative stress, inflammation, and liver fat accumulation (lipotoxicity) [3,61]. Therefore, reactive species rise and their contact with lipids, particularly polyunsaturated fatty acids, results in hydroperoxides (LOOH) and as a secondary product of MDA, this process is called lipoperoxidation [62]. Hence, MDA levels are a biomarker of a rise in hydroperoxides production, lipid peroxidation, and the rise of reactive species [62]. Once these hydroperoxides are generated, antioxidant enzymes such as GPx and CAT catalyze the reduction of H_2_O_2_ [62]. Accordingly, the livers of the H rats showed lipid peroxidation (↑MDA) and, consequently, the rise in reactive species switch on antioxidant mechanisms (↑GSH and *Hmox1*). HO-1 is an enzyme with antioxidant defense functions; it catalyzes heme to iron, carbon monoxide, and biliverdin [63]. HO-1 induction is regulated by different stimuli, its overexpression can be upregulated by the JNK pathway [64], related to insulin resistance and the overexpression of *Tnfa.* Meanwhile, HBL showed a lower expression of *Tnfa* than those in the S group. A decrease in TNFalpha has been related to lower activation of NFkB [65]. However, there are no reported studies where the decreased expression of TNFalpha has been related to a pathological condition or to a decrease in the immune system.

Nrf2 has an important dual role in NAFLD progression [50,66]. Under stress conditions, Nrf2 has canonical and non-canonical activation mechanisms [66]. In the canonical mechanism, Keap-1 senses the reactive species and releases Nrf2 in the cytoplasm, ready to translocate to activate the enzymatic (HO-1, GPx, CAT) and non-enzymatic (GSH) antioxidant defense systems by binding in the nucleus to ARE (antioxidant response element) [9,50]. The non-canonical pathway is p62-dependent; it is usually related to chronic Nrf2 activation and the development of chronic diseases such as diabetes, NAFLD, and cancer [66,67]. Then, the higher expression of *Nfe2l2* mRNA in the H group compared to that of S could be related to the activation of Nrf2 by the non-canonical pathway. Despite Nrf2 activation by the non-canonical pathway had been related to metabolic alterations development in their late stages, more studies are needed to explore the role of Nrf2 in early stages of NAFLD (steatosis) [66,67].

Plant extracts, probiotics, and prebiotics had been useful in NAFLD treatment by reducing inflammation and increasing the antioxidant defense system, particularly GSH levels [68]; and bean leaves had shown an important content of polyphenols and dietary fiber besides preventing obesity-related comorbidities [6,7,8]. This is the first study that evaluates the effect of bean leaves on NAFLD. HBL diet showed a protective effect against steatosis grade I. Formerly, the combination of bean leaves supplementation and RFP (7-h daytime-restricted-feeding protocol) had ameliorated insulin resistance and liver fat accumulation in a murine high-fat/high-fructose model [8]. Steatosis is asymptomatic and curable, so it is important to focus research on its prevention and treatment, considering local customs and habits [59,60].

PPARα which is a main regulator of lipid metabolism, regulates many genes involved in lipid metabolism, fatty acid uptake, oxidation (mitochondrial and peroxisomal), and triglyceride turnover [41]. PPARα also modulates inflammation mediated by direct binding to the p65 subunit of NFκB [41]. NFκB is a transcriptional regulator of TNFα [69], and PPARα is upregulated by Nrf2 [10,12]. *Ppara* expression in HBL rats increased compared to H rats. These findings suggest that bean leaves have a protective role against hepatic lipid accumulation. Previously, the supplementation with bean leaves for 6 weeks had a 54% increase in SCFA production due to the fermentation of the bioactive compounds present in bean leaves [7]. SCFA have positive effects in metabolic disease prevention and are able to interact with different tissues. Interestingly, some studies have suggested that SCFA can interact with hepatic PPARs; specifically, they can active PPARα expression reducing lipid accumulation by increasing lipid β-oxidation in the liver and adipose tissue [70], but the mechanism is not clearly elucidated [2]. Future research should evaluate the role of the liver–gut axis in the prevention of NAFLD, highlighting the effect of bean leaves on SCFA production and their possible interaction with PPARα in the liver.

The liver is mainly responsible for GSH homeostasis in the body [68,71]. Glutathione, an important thiol redox agent, is mainly synthesized in macrophages in different tissues including blood, and the liver exports it into blood and bile. The increase in serum oxidized glutathione (GSSG) induces hepatic gamma-glutamyl transpeptidase (GGT), and GGT leads to the conversion of GSSG into GSH. Interestingly bean leaves supplementation increased serum GSH enhancing antioxidant capabilities in the body but without statistical changes in liver GSH. These could be related to less need for GSH in order to lower lipid peroxidation due to less reactive spices (↓MDA) in the liver due to the high capacity of the liver for GSH efflux through its basolateral and apical poles to maintain interorgan homeostasis of GSH by rising serum GSH [68].

Inducers of HO-1, such as curcumin, berberine, and resveratrol, had been studied as a possible treatment of NAFLD, because HO-1 overexpression had enhanced lipogenesis and collagen production [63,72]. Likewise, bean leaves induce a higher expression of Hmox1 mRNA in HBL and SBL groups. Additionally, this overexpression in bean leaves supplemented groups could be related to a higher intake of iron, based on the potential effect that bean leaves showed in a previous study, where bean leaves were used as a treatment for anemia rising up hemoglobin levels because of its iron bioavailability, [6]. Therefore, bean leaf intake could increase the requirement of HO-1 to catalyze heme [63].

GSH, HO-1, and other antioxidant enzymes are regulated by Nrf2. Nrf2 has shown potential as a therapeutical target in NAFLD progression [9]. Nrf2 activates PPARα, leading to β oxidation and ameliorated fat lipid accumulation in the liver [10,11].

Additionally, due to the interaction of dietary antioxidants as polyphenols with Keap-1 (canonical pathway), Nrf2 is able to induce antioxidant defense systems and delay inflammatory response mediated by NFκB [10,12]. Therefore, SBL rats showed a higher nuclear translocation; this suggested an antioxidant activation that needs to be further explored. However, HBL and SBL rats exhibited reduced expression of Nfe2l2 mRNA, possibly owing to the rise of GSH levels in serum, less hepatic fat accumulation, and lower lipid peroxidation. In light of these findings, bean leaves appear promising as a dietary alternative in the prevention of metabolic alterations, particularly steatosis and hepatic lipotoxicity (↓hepatic triglycerides and ↓MDA).

The expression of Ppara mRNA and the expression of Nfe2l2 mRNA did not seem to be regulated by bean leaves. Nrf2 is not the only regulator of PPARα, glucocorticoids, dietary fatty acids, eicosanoids, endocannabinoids, and (lyso)phospholipids [73]. Meanwhile, Nfe2l2 transcription is downregulated during oxidative stress in the liver by control nonderepressible 2 (GCN2) [74], further analyses are needed to determine if the decreased expression of Nfe2l2 in bean leaves supplementation is due to GCN2.

## 5. Conclusions

NAFLD is a worldwide public health problem, and understanding its complex pathology is key to the development of prevention strategies and treatments, particularly at the early stage of steatosis, which is reversible. Insulin resistance, hyperlipemia, and obesity play an important role in lipotoxicity in fatty liver development; prevention strategies should also approach them. Bean leaves supplementation such as plant-based foods interventions had shown beneficial effects in the treatment and prevention of metabolic disorders related to a long-term high-fat/high-fructose diet due to their content of bioactive compounds. Bean leaves are a source of bioactive compounds such as iron, dietary fiber, and polyphenols. As with other plant-based interventions, they are a low-cost alternative for nutritional interventions; moreover, beans are widely cultivated around the world. Supplementation with 10% of bean leaves in a high-fat/high-fructose diet ameliorated the insulin resistance and compensatory hyperinsulinemia; prevented the development of steatosis, and lipid peroxidation, activated the antioxidant defense system, and showed an anti-inflammatory effect, reducing hepatic lipotoxicity. Further studies are needed to deeply understand the mechanism of action of bean leaves supplementation in the prevention of metabolic alterations.

## Figures and Tables

**Table 1:** Composition of experimental diets.

Diet	Energy (kcal/g)	Protein (g/100 g)	Fat (g/100 g)	Carbohydrate (g/100 g)	Fiber (g/100 g)	Ingredients
S	3.4	25.3	11.4	48.2	5.3	RLC 5001^®^
SBL	3.6	26.4	11.9	51.8	5.1	RLC 5001^®^, bean leaves, calcium caseinate, soy oil
H	4.6	22.3	43.2	44.4	3.6	RLC 5001^®^, lard, fructose, calcium caseinate, wheat bran
HBL	4.8	22.1	43.4	49.6	3.7	RLC 5001^®^, lard, fructose, calcium caseinate, bean leaves

RLC = Rodent Laboratory Chow, S = standard diet, SBL = S + 10% bean leaves, H = high-fat/high-fructose diet, HBL = H + 10% bean leaves.

**Table 2:** List of primers for real-time PCR analysis.

*Gene* (Bank Number)	Primer Sequence 5′ to 3′
*Sod2* (NM_017051.2)	Fwd: TGGACAAACCTGAGGCCTAA Rev: GACCCAAAGTCACGCTTGATA
*Ywhaz* (NM_013011.4)	Fwd: TTGAGCAGAAGACGGAAGGT Rev: GAAGCATTGGGGATCAAGAA
*Tnfa* (AY427675.1)	Fwd: TGGGCTGTACCTTATCTACTCC Rev: GGCTGACTTTCTCCTGGTATG
*Nfe2l2* (BC061724.1)	Fwd: CAGAAGGAACAGGAGAAGGC Rev: TCAACGTGGCTGGGAATATC
*Ppara* (NM_013196.2)	Fwd: GGGTCATACTCGCAGGAAAG Rev: ACCTGGTCATTCAAGTCCAAG
*Hmox1* (NM_012580.2)	Fwd: ACAGAGGAACACAAAGACCAG Rev: GAGAAGGCTACATGAGACAGAG

*Sod2* = Superoxide dismutase 2, *Ywhaz =* Tyrosine 3-monooxygenase/tryptophan 5-monooxygenase activation protein zeta, *Tnfa* = Tumor necrosis factor-alpha, *Nfe2l2 =* Nuclear factor erythroid 2-related factor 2, *Ppara* = Peroxisome proliferator-activated receptor alpha, *Hmox1* = Heme oxygenase 1.

**Table 3:** Effect of bean leaves in body measurements.

	S	SBL	H	HBL
Total body weight gain (g)	293.31 ± 10.67	275.39 ± 12.7	344.61 ± 15.3 *	315.83 ± 19.11
Body length (cm)	24.74 ± 0.85	23.68 ± 0.61	24.6 ± 0.27	23.38 ± 0.44 ^#^
Abdominal circumference (cm)	21.67 ± 0.47	20.93 ± 0.57	22.98 ± 0.54	21.36 ± 0.65
Thoracic circumference (cm)	19.39 ± 0.26	18.59 ± 0.46	20.00 ± 0.48	18.61 ± 0.39 ^#^
AC/TC ratio	1.12 ± 0.03	1.13 ± 0.05	1.16 ± 0.04	1.15 ± 0.01
BMI (g/cm^2^)	0.80 ± 0.04	0.86 ± 0.04	0.90 ± 0.02	0.89 ± 0.04

Values represent the mean ± SEM (*n* = 9). ANOVA post hoc Dunnet’s test was performed to compare groups versus S, * *p* ≤ 0.05. Student’s *t*-test was performed to compare H versus HBL, ^#^ *p* ≤ 0.05. S = standard diet, SBL = S + 10% bean leaves, H = high-fat/high-fructose diet, HBL = H + 10% bean leaves, AC = abdominal circumference, TC = thoracic circumference, BMI = body mass index.

**Table 4:** Food, energy and water intake.

	S	SBL	H	HBL
Food intake (g/day)	38.0 ± 2.0	37.9 ± 1.7	38.8 ± 1.4	38.7 ± 2.2
Total food intake (g)	494.7 ± 26.1	492.3 ± 22.1	503.9 ± 18.7	503.0 ± 28.0
Energy intake (kcal/day)	129.4 ± 6.8	136.3 ± 6.1	170.6 ± 6.3 *	178.0 ± 9.9 *
Total energy intake (kcal)	1681.9 ± 88.9	1772.2 ± 79.4	2191.6 ± 77.1 *	2313.8 ± 129.0 *
Water intake (mL/day)	55.7 ± 2.8	55.2 ± 3.2	44.8 ± 4.2	41.0 ± 2.6 *
Total water intake (mL)	723.7 ± 36.7	717.8 ± 41.5	583.5 ± 55.3	533.4 ± 34.8 *

Values represent the mean ± SEM (*n* = 9). ANOVA post hoc Dunnet’s test was performed to compare groups versus S, * *p* ≤ 0.05. Student’s *t*-test was performed to compare H versus HBL, (*p* ≤ 0.05), not statistically different. S = standard diet, SBL = S + 10% bean leaves, H = high-fat/high-fructose diet, HBL = H + 10% bean leaves.

**Table 5:** Effect of bean leaves in glucose metabolism.

	S	SBL	H	HBL
Glucose (mg/dL)	130.67 ± 6.69	126.38 ± 5.60	129.91 ± 9.17	125.02 ± 9.10
Insulin (pM)	93.97 ± 9.62	112.51 ± 10.98	227.19 ± 38.03 *	177.47 ± 20.74 *
HOMA-IR	4.29 ± 0.47	4.81 ± 0.40	9.29 ± 1.63 *	7.71 ± 1.07
HOMA-β	32.1 ± 4.0	47.0 ± 6.9	115.1 ± 26.4 *	81.7 ± 13.4

Values represent the mean ± SEM (*n* = 9). ANOVA post hoc Dunnet’s test was performed to compare groups versus S, * *p* ≤ 0.05. Student’s *t*-test was performed to compare H versus HBL (*p* ≤ 0.05), not statistically different. S = standard diet, SBL = S + 10% bean leaves, H = high-fat/high-fructose diet, HBL = H + 10% bean leaves, HOMA-IR = homeostatic model assessment for insulin resistance, HOMA-β = homeostatic model assessment for pancreatic β cell function.

**Table 6:** Effect of bean leaves in lipid profile.

	S	SBL	H	HBL
Total cholesterol (mg/dL)	69.95 ± 2.93	64.87 ± 2.31	87.88 ± 2.82 *	78.64 ± 2.99 ^#^
Triglycerides (mg/dL)	104.61 ± 6.55	99.97 ± 9.17	165.00 ± 12.03 *	147.99 ± 9.59 *
VLDL-c (mg/dL)	20.92 ± 1.31	19.99 ± 1.83	33.00 ± 2.41 *	29.60 ± 1.92 *
LDL-c (mg/dL)	13.33 ± 2.05	14.25 ± 1.72	12.80 ± 1.18	9.90 ± 1.00
OxLDL (ng/mL)	28.48 ± 1.71	30.43 ± 1.42	32.03 ± 1.74	32.83 ± 1.40
HDL-c (mg/dl)	46.31 ± 1.93	43.82 ± 1.00	54.43 ± 1.94 *	49.46 ± 2.03
Triglycerides/ HDL-c ratio	4.17 ± 0.70	3.24 ± 0.57	5.26 ± 1.06	5.04 ± 0.92

Values are mean ± SEM (*n* = 9), comparison against S was analyzed by ANOVA post hoc Dunnet * *p* ≤ 0.05, and differences between H and HBL was analyzed using Student’s *t*-test (^#^ *p* ≤ 0.05), not statistically different. S = standard diet, SBL = S + 10% bean leaves, H = high-fat/high-fructose diet, HBL = H + 10% bean leaves, VLDL-C = very low-density lipoprotein, LDL-C = low-density lipoprotein, OxLDL = oxidized low-density lipoprotein, HDL-C = high-density lipoprotein.

**Table 7:** Prevention of liver steatosis in high-fat/fructose diet.

	Steatosis Grade		
Group	0 (%)	I (%)	Findings
S	100	0	Adequate histoarchitecture without damage
SBL	100	0	Adequate histoarchitecture without damage
H	0	100 *	Macrovesicular steatosis < 33%, centrilobular
HBL	100	0	Microvesicular steatosis < 5%, centrilobular

Scoring the steatosis grade according to Brunt. Statistical differences were analyzed by Chi-squared test, * *p* < 0.05. S = standard diet, SBL = S + 10% bean leaves, H = high-fat/high-fructose diet, HBL = H + 10% bean leaves.

**Table 8:** Effect of bean leaves (H + BL) in liver function.

	S	SBL	H	HBL
AST (U/L)	189.62 ± 11.89	180.57 ± 10.73	179.25 ± 12.50	185.36 ± 13.14
ALT (U/L)	87.62 ± 5.88	83.00 ± 4.59	73.06 ± 5.38	80.36 ± 3.78
AST/ALT ratio	4.34 ± 0.91	4.09 ± 0.82	5.79 ± 1.58	4.36 ± 0.91
Total protein (g/dL)	6.34 ± 0.08	6.29 ± 0.10	6.52 ± 0.08	6.59 ± 0.15
Albumin (g/dL)	4.23 ± 0.23	4.16 ± 0.24	4.28 ± 0.22	4.25 ± 0.24
Globulin (g/dL)	2.11 ± 0.26	2.13 ± 0.19	2.24 ± 0.20	2.34 ± 0.20
A/G ratio	9.09 ± 4.09	5.52 ± 1.61	6.19 ± 1.99	5.07 ± 1.53
CRP (mg/mL)	0.32 ± 0.05	0.30 ± 0.04	0.35 ± 0.02	0.34 ± 0.02

Values are mean ± SEM (*n* = 9), comparison against S was analyzed by ANOVA post hoc Dunnet (*p* ≤ 0.05 and comparison between H and HBL was analyzed with Student’s *t*-test (*p* ≤ 0.05). Not statistically different. S = standard diet, SBL = S + 10% bean leaves, H = high-fat/high-fructose diet, HBL = H + 10% bean leaves, AST = aspartate aminotransferase, ALT = alanine aminotransferase, A = albumin, G = globulin, CRP = C-reactive protein.

## Data Availability

The data will be available by contacting the corresponding author.
